# Utilization of Norway’s Emergency Wards: The Second 5 Years after the Introduction of the Patient List System 

**DOI:** 10.3390/ijerph110303375

**Published:** 2014-03-21

**Authors:** Ursula S. Goth, Hugo L. Hammer, Bjørgulf Claussen

**Affiliations:** 1 Faculty of Education and International Studies, Oslo and Akershus University College of Applied Sciences, 0130 Oslo, Norway; 2 Faculty of Technology, Art and Design, Oslo and Akerhus University College of Applied Sciences, 0130 Oslo, Norway; E-Mail: hugo.hammer@hioa.no; 3 Faculty of Medicine, University of Oslo, 0316 Oslo, Norway; E-Mail: bjorgulf.claussen@medisin.uio.no

**Keywords:** primary health care, utilization, immigrant, emergency ward, general practitioner, patient list system, Norway

## Abstract

Utilization of services is an important indicator for estimating access to healthcare. In Norway, the General Practitioner Scheme, a patient list system, was established in 2001 to enable a stable doctor-patient relationship. Although satisfaction with the system is generally high, people often choose a more accessible but inferior solution for routine care: emergency wards. The aim of the article is to investigate contact patterns in primary health care situations for the total population in urban and remote areas of Norway and for major immigrant groups in Oslo. The primary regression model had a cross-sectional study design analyzing 2,609,107 consultations in representative municipalities across Norway, estimating the probability of choosing the emergency ward in substitution to a general practitioner. In a second regression model comprising 625,590 consultations in Oslo, we calculated this likelihood for immigrants from the 14 largest groups. We noted substantial differences in emergency ward utilization between ethnic Norwegians both in rural and remote areas and among the various immigrant groups residing in Oslo. Oslo utilization of emergency ward services for the whole population declined, and so did this use among all immigrant groups after 2009. Other municipalities, while overwhelmingly ethnically Norwegian, showed diverse patterns including an increase in some and a decrease in others, results which we were unable to explain.

## 1. Introduction

Equity in health care might be understood as the absence of systematic health disparities in the population [1]. In order to increase equity, Norway introduced the “Regular General Practitioner scheme” (GP Scheme) in 2001, a reform aiming at comprehensive stability and efficiency in the general practitioner–patient relationship, especially important for people suffering from recurring or complex medical problems. This patient list system is anchored at the municipal level, and entitles every resident to an assigned GP on a voluntary basis [2]. 

The concept of emergency primary care, offered out of office hours, is for patients to get access to immediate medical care and receive essential medical diagnostics and treatment for acute illness or injuries. 

In Norway the emergency ward (EW) is part of the primary health care services. The concept of emergency primary care, offered out of office hours, is for patients to get access to immediate medical care [3]. In larger cities the EW is situated in a designated location in the city center and served by the GPs but in the three largest cities—Oslo, Bergen, and Trondheim—physicians on duty are employed by the municipality on fixed wages. GPs on EW duty are paid by the ordinary National Health Insurance tariff for private general practice [3]. 

Within the GP Scheme, a GP is assigned to all immigrants who possess a permit to stay over six months. Still, many immigrants prefer the EW instead [4]. Also many others who could and should visit their assigned GP still use the EWs frequently because that is an easier-access option instead of the GP [5]. This abuse of the EW for routine care represents an inferior solution for the patient, disrupts continuity of care, and increases costs for the municipality. But this excessive use might also indicate barriers impeding successful promotion of health care and disease prevention. With an increasing diversity within the population, this poses new and additional challenges to the national health system. As a multi-cultural society today, Norway has 547,000 immigrants with 108,000 descendants. Immigrants represent 13.1% of the population [6]. Two out of ten immigrants have resided longer than 20 years, and 4 of 10 have been in Norway for less then four years [7]. Oslo (the capital of Norway), as well as Bergen and Drammen, have the highest density of immigrants. Scandinavian research indicates a diverging picture regarding utilization of EW services by immigrants compared to non-migrants [8,9,10,11]. A Norwegian study concluded that high rates of EW use among immigrants are related to inadequate access to GP services, suggesting that new residents are not well informed about the organizational structure of the primary health sector [11]. Another study has demonstrated that immigrants use the EW more often than non-migrants, and that the differences in EW use among immigrant groups are smaller than the differences between genders in these groups, confirming that the duration of residence influences the likelihood of visiting the EW and that this impact is greatest during the first years after arrival [8].

The aim of this study was to examine the pattern of contact of the GP Scheme system ten years after its introduction, addressing two questions:

1. What have the utilization patterns of EW in large and small municipalities been for the residents of Norway five years after the introduction of the GP Scheme?
2. What have immigrants’ utilization patterns in Oslo been ten years after the introduction of the system?

## 2. Methods

**Figure 1:**
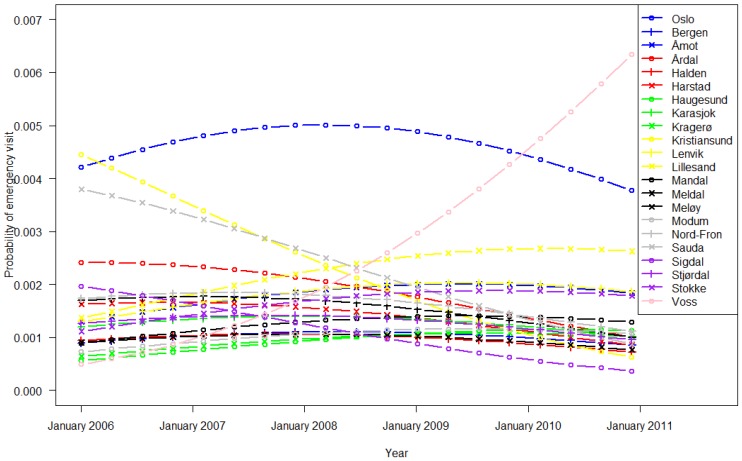
EW utilization during office hours (Monday to Friday 9:00 a.m. to 3:00 p.m.) across calendar years and municipalities.

**Figure 2:**
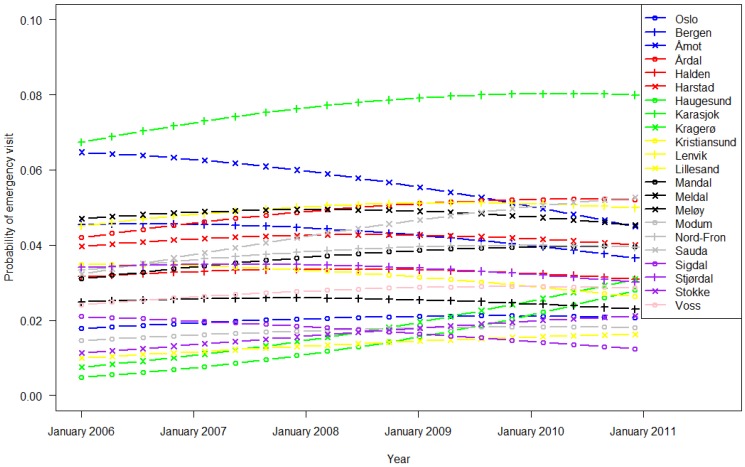
EW utilization outside office hours (Monday to Friday 3:00 p.m. to 9:00 a.m. and during weekends) across calendar years and municipalities.

**Figure 3:**
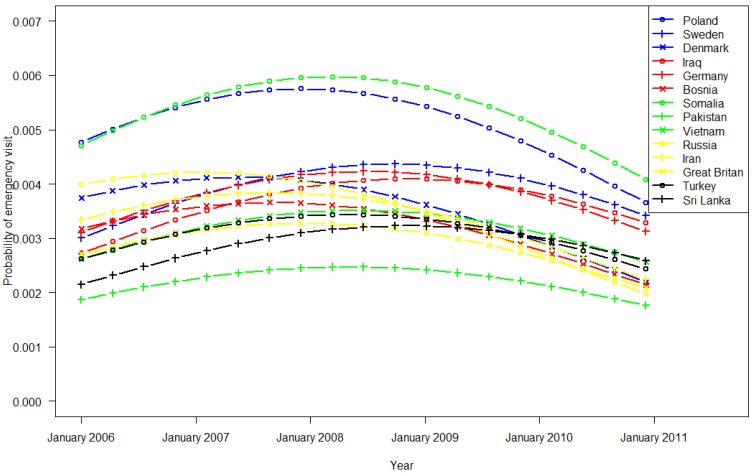
EW utilization by immigrants (country of origin) in Oslo during GPs office hours (Monday to Friday 9:00 a.m. to 3:00 p.m.).

**Figure 4:**
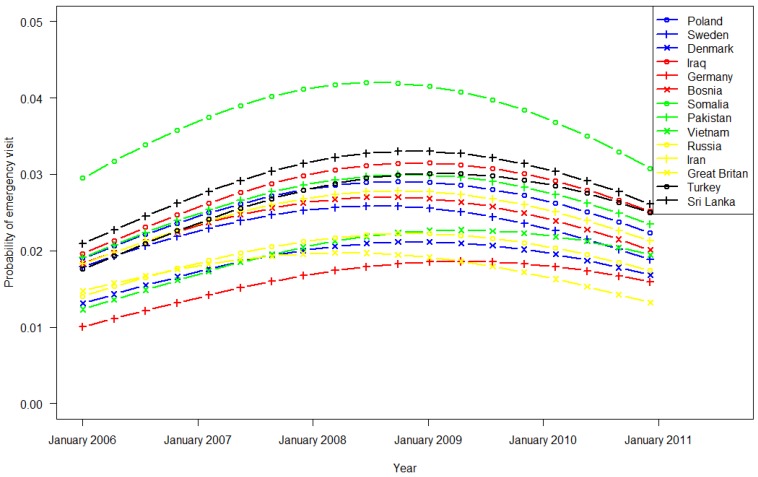
EW utilization by immigrants (country of origin) in Oslo outside GPs office hours (Monday to Friday 3:00 p.m. to 9:00 a.m. and during weekends).

In 2010 the population of Norway comprised 4,858,199 persons [12]. Our dataset of all 40 million consultations in Norway (from these nearly 5 million inhabitants) in the years 2006, 2007, 2009, and 2010 was delivered by the Norwegian Labour Welfare Administration (NAV). Data for 2008 were corrupt and could not be used, therefore in Figure 1, Figure 2, Figure 3 and Figure 4 data for this year was extrapolated. 

In our dataset we divided all consultations in two groups, the first at the individually assigned GP and second consultations at the EW. Variables stringed to the individual person were gender, age, country of birth, and municipality of residence. All persons and their variables were obtained de-identified from Statistics Norway, responsible for the construction of the database. 

All persons under the age of 19, a little over one million inhabitants, were excluded. To analyse the impact of various factors on the probability of consulting an EW, linear mixed logistic regression models were used. The Deviance Information Criterion (DIC) was used as guidance for inclusion of variables [13]. To explore our two research questions, the data analyses were divided in a representative sample of the various municipalities and a sample with maximum variation of immigrants. In order to run regressions with our program, we had to restrict the sample as explained below.

### 2.1. The First Study Population: Various Municipalities in Norway

The first study population comprised 2,609,107 consultations by 238,012 individuals. Individuals included were all legal residents of Norway who had contacted their GP or the EW at least once during the four years. We chose 22 of the 224 municipalities representing all counties and differing in both size and location. For the two biggest municipalities, Oslo and Bergen, we limited the population to a random sample of 400,000 individuals in the regression analyses. This limitation in size allowed us to perform regression analyses for all 22 municipalities simultaneously. On the other hand, in the descriptive statistical analysis, all individuals in Oslo and Bergen were included. The first analysis concerned the utilization patterns of EWs during and outside of GP office hours, fitting one regression model for EW usage during office hours and one outside office hours. By analysing utilization for various municipalities only ethnic Norwegians were included. We adjusted for year, year^2, age and age^2, because we had a hypothesis that the association between consultations and these two variables might be curvilinear. Further we adjusted for gender, municipality, and the interaction between municipality and year. All the above variables improved the DIC. “Year” refers to the time of consultation (year and date, e.g., 21 May 2010). Interaction between age^2 and municipalities also slightly improved DIC for the EW usage outside office hours, but were not included since the comparison with the model for EW usage in office hours became harder. Also, excluding this interaction did not change interpretations of the model. No other two-way or higher-order interaction improved the DIC of the models. 

### 2.2. The Second Study Population: Various Groups of Immigrants in Oslo

The study population included the 14 largest groups of immigrants in Norway in 2007. Those were, in descending order, Pakistan, Sweden, Iraq, Somalia, Denmark, Poland, Vietnam, Bosnia, Iran, Turkey, Germany, Serbia, Sri Lanka, United Kingdom, and Russia (Statistics Norway). The database includes data up to December 31st 2010. At that time the ranking changed, as Poland became the leading source of immigrants to Norway. 

Here, we examined immigrants’ use of the EW in Oslo, as it has both the largest diversity of immigrants and a round-the-clock EW. These 14 largest groups in 2006 [12] comprised 625,590 consultations during the four years. As explanatory variables for EW use, we adjusted for gender, country of birth, duration of residence in Norway, year, year^2, age, age^2, duration^2, and the interaction between birth country and year and duration of residence. By including year^2, age^2, and duration^2, the DIC of the model improved. No other two-way or higher-order interaction improved the DIC of the model. The regression analyses were performed using the INLA package in the statistical program R [14]. 

### 2.3. Ethics and Trial Registration

The Norwegian Regional Committee for Medical and Health Research Ethics approved this project and the construction of the database with the clearance number 2010/537. The approval was based on the acceptance of the owners of the registries, the Directorate of Health, the Norwegian Labour and Welfare Administration, and the Ministry of Health and Care services. 

## 3. Results

The pattern of contact with EW varied between the 22 municipalities for the years 2006, 2007, 2009, and 2010 (Table 1). Outside GP office hours, EW utilization ranged from 3% to 15%. During office hours, Oslo had the highest frequency of contact with EWs, but outside of office hours, most municipalities had greater frequencies than Oslo. The 22 municipalities varied widely with regard to the number of inhabitants, but in some other respects were similar, such as in the proportion of female consultations (56 to 64%) and the mean age (52 to 59 years). Regression model 1 showed that the pattern of EW utilization was diverse (Table A1 in the appendix). Age^2 and year^2 differed significantly from 0, showing a nonlinear relationship between these variables and the probability of seeking EW. As the null hypothesis for those parameters is zero, we actually observed nonlinear relations. The rate of contact with EWs during office hours was significantly lower throughout the country compared with Oslo. Outside of office hours, Bergen had significantly greater likelihood of contact with EW, whereas the small town of Haugesund had a smaller probability than Oslo. 

Figure 1 and Figure 2 show the parameters of the regression model for 2006 to 2010, demonstrating a constant use of EWs during GP office hours, except for a significant increase in the municipality of Voss since 2007. 

**Table 1:** Demographic data of primary health care consultations - EW contacts during and outside office hours in 22 municipalities. Number of individual patients, number of consultations and proportion of EW consultations for the time period 2008–2010 in selected Norwegian municipalities (95% CI).

Municipalities	Number of Individual Patients	Total Number of Consultations	Proportion of EW Consultations in Selected			
			During Office Hours		Outside Office Hours	
Oslo	294,384	3,146,083	0.69	(0.68–0.69)	4.42	(4.41–4.44)
Bergen	150,102	1,625,573	0.21	(0.21–0.22)	8.10	(8.07–8.14)
Åmot (Hedmark)	2,983	30,555	0.43	(0.37–0.49)	10.42	(10.1–10.7)
Årdal	3,777	45,553	0.39	(0.34–0.43)	8.96	(8.74–9.18)
Halden	17,827	207,760	0.18	(0.16–0.19)	6.77	(6.68–6.86)
Harstad	15,720	179,900	0.28	(0.26–0.30)	8.01	(7.91–8.12)
Haugesund	18,883	182,629	0.13	(0.12–0.15)	3.28	(3.21–3.35)
Karasjok	1,828	24,396	0.39	(0.32–0.46)	15.22	(14.8–15.6)
Kragerø	7,041	87,263	0.13	(0.12–0.15)	4.03	(3.92–4.14)
Kristiansund	13,056	99,336	0.18	(0.16–0.20)	5.77	(5.65–5.89)
Lenvik	7,631	99,332	0.41	(0.37–0.44)	9.41	(9.26–9.56)
Lillesand	5,951	73,240	0.29	(0.25–0.32)	2.94	(2.84–3.04)
Mandal	9,076	109,139	0.25	(0.23–0.28)	7.64	(7.51–7.77)
Meldal	2,910	35,723	0.23	(0.19–0.27)	4.89	(4.70–5.08)
Meløy	4,464	52,150	0.21	(0.18–0.25)	9.23	(9.02–9.44)
Modum	7,910	68,743	0.15	(0.13–0.18)	3.66	(3.54–3.78)
Nord-Fron	4,176	52,408	0.30	(0.26–0.33)	7.21	(7.02–7.39)
Sauda	3,231	32,284	0.49	(0.42–0.55)	9.04	(8.77–9.30)
Sigdal	2,416	25,430	0.12	(0.09–0.16)	3.05	(2.87–3.23)
Stjørdal	14,059	178,994	0.23	(0.21–0.25)	6.45	(6.35–6.54)
Stokke	6,852	74,787	0.23	(0.20–0.26)	3.66	(3.54–3.77)
Voss	8,777	91,962	0.51	(0.47–0.55)	5.13	(5.01–5.25)

Regression model 1, which describes the contact outside of GP office hours, indicates probabilities between 0.01 and 0.05, except in Karasjok municipality, which lies far north inland, with probabilities between 0.07 and 0.08 (further information in Appendix Table A1). Compared with Oslo, most municipalities experienced greater use of EWs outside office hours, as shown in Figure 2.

Regression model 2 was based on data from immigrants belonging to the 14 largest groups in 2006, comprising 625,590 consultations by 53,209 patients. During office hours, the impact of birth country was significantly greater for Somalia and smaller for the other countries compared with Poland (Figure 3). All variables were statistically significant in the regression model. Outside office hours, as shown in Figure 4, Pakistani immigrants had the highest use of EW services, and German immigrants had the lowest use. The frequency of EW contact overall rose in 2006 and 2007 and declined in 2009 and 2010 during and outside office hours. These patterns varied significantly between ethnic groups (analysis is shown in Table A2 in the Appendix).

## 4. Discussion

EW use varied widely among ethnic Norwegian inhabitants in the 22 municipalities (Table 1, Figure 1 and Figure 2). The frequent use in Oslo and Bergen is likely attributed to the round-the-clock opening hours. In recent years, Voss has experienced increased participation in alpine skiing and extreme sports activities, which might explain the rise in EW usage during office hours. Otherwise, we did not find any other obvious patterns for rural/urban or small/bigger municipalities. These trends regarding EW use have been constant for the past five years (Figure 1 and Figure 2) despite introduction of the GP scheme ten years ago to increase the continuity of care [2]. As in previous studies we observed higher utilization rates among immigrants compared to non-migrants [15]. Despite the aim of the GP Scheme, EW use differed between the 14 largest immigrant groups, adjusted for duration of stay in Norway. Based on the utilization 5–10 years after the introduction of the GP Scheme, there has been no reduction in EW use, or consequently, greater contact with the assigned GP.

Immigrants from Poland and Somalia had the highest use of EW services during GP office hours, after controlling for age and duration of residence. The propensity for immigrant groups to use the EW during office hours varied independently of country of birth (remote or in close proximity to Norway), indicating that health system barriers are in place, and that organizational adjustments must be made to ensure health services to the diverse strata in the population. Encountering obstacles of access might cause such barriers. These obstacles might be related to formal barriers, associated with the organization of the GP system, and informal barriers that are formed due to problems with language and communications, sociocultural factors, and its relative newness [11,13,16]. Contrary to what we expected, the probability of visiting an EW outside office hours (3 p.m.–9 a.m.) increased for all immigrant groups. Thus, research must focus on this phenomenon, and efforts need to be made to ensure equitable health services. Notably, despite the introduction of the GP Scheme and the political focus on accessibility, we noted a rise in EW use over time.

## 5. Strengths and Limitations

The strength of the study was the complete accounting of all contacts to the EW or GP over a given period in a selection of 22 municipalities, except for Oslo and Bergen, where a fraction of the whole were used for analysis. All data are based on a reimbursement registry. In logistic/multinomial regressions, ten samples per independent variable were considered in the model. Correlations between the independent variables in excess of 0.4 (absolute value) indicate multicolinearity. Here, no correlations were above 0.23. The database was reliable but the study was limited by only considering residents who contacted a GP or EW during the four years included in the study. Due to the lack of socio-economic variables and the specific diagnoses, no further adjustments could be made.

## 6. Conclusions

- Our results show that no consistence in utilization in the time period of five to ten years after the introduction of the GP Scheme.
- Data on utilization, based on health care needs and access to the primary health care system, show that equity in health care services is not achieved.
- Access to health care for immigrants in Oslo has eased in recent years.
- In contrast, Norwegian-born residents had diverse patterns, with increased EW use in certain municipalities and lower use in others—results that we cannot explain. Further research is necessary to examine these patterns.

## Appendix

**Table A1:** Immigrants in Oslo: Estimated linear regression parameters for the impact of year 2006, 2007, 2009, and 2010, duration of residence, age, gender, and birth country on the probability of contacting EW outside and during GP office hours.

Independent Variables	Estimated Values Outside GP Office Hours	Estimated Values During GP Office Hours
Year^2	−0.0588 ***	−0.05084 ***
Duration^2	0.00037 ***	0.00082 ***
Age	−0.09479 ***	−0.08728 ***
Age^2	0.00085 ***	0.00083 ***
Gender	−0.12388 ***	−0.22114 ***
Sweden	−0.10503 *	−0.23468 **
Denmark	−0.25849 ***	−0.35674 ***
Iraq	0.27865 ***	−0.14675
Germany	−0.38033 ***	−0.23454 *
Bosnia	−0.06286	−0.34259 **
Somalia	0.63588 ***	0.35259 ***
Pakistan	0.15933 ***	−0.60367 ***
Vietnam	−0.23191 ***	−0.31261 **
Russia	−0.06656	−0.20094
Iran	0.03515	−0.26020 **
Great Britain	−0.37190 ***	−0.44712 ***
Turkey	0.11838 **	−0.35636 ***
SriLanka	0.20439 ***	−0.28970 **
Poland × Year	0.86275 ***	0.66253 ***
Sweden × Year	0.83982 ***	0.74292 ***
Denmark × Year	0.88063 ***	0.60810 ***
Iraq × Year	0.88199 ***	0.75471 ***
Germany × Year	0.92472 ***	0.7178 ***
Bosnia × Year	0.84885 ***	0.63648 ***
Somalia × Year	0.83841 ***	0.68780 ***
Pakistan × Year	0.87247 ***	0.70436 ***
Vietnam × Year	0.92511 ***	0.71254 ***
Russia × Year	0.87348 ***	0.57348 ***
Iran × Year	0.86131 ***	0.63538 ***
GreatBritain × Year	0.80594 ***	0.66423 ***
Turkey × Year	0.90340 ***	0.70189 ***
SriLanka × Year	0.87647 ***	0.75472 ***
Poland × Duration	−0.0393 ***	−0.07540 ***
Sweden × Duration	−0.03505 ***	−0.07534 ***
Denmark × Duration	−0.02564 ***	−0.06712 ***
Iraq × Duration	−0.00326	−0.04688 ***
Germany × Duration	−0.02135 ***	−0.06813 ***
Bosnia × Duration	−0.03629 ***	−0.04885 ***
Somalia × Duration	0.00662	−0.02040 **
Pakistan × Duration	−0.01563 ***	−0.03494 ***
Vietnam × Duration	−0.03241 ***	−0.04744 ***
Russia × Duration	0.00069	−0.03123 **
Iran × Duration	−0.02405 ***	−0.04225 ***
Great Britain × Duration	−0.02920 ***	−0.05311 ***
Turkey × Duration	−0.02397 ***	−0.05025 ***
SriLanka × Duration	−0.02674 ***	−0.02897 ***

Notes: ***** *p* < 0.05, ****** *p* < 0.01, ******* *p* < 0.001; Female = 1, Male= −1; Reference is Poland.

**Table A2:** Norwegian born inhabitants. Estimated linear regression parameters for the impact of year 2006, 2007, 2009 and 2010, age, gender and municipality on the probability of seeking EW across 22 selected municipalities outside and during GP office hours.

Independent Variables	Estimate Value	
	Outside GP Office Hours	During GP Office Hours
Year^2	−0.01368 ***	−0.01651 ***
Age	−0.00071 ***	−0.00119 ***
Age^2	0.00088 ***	0.00137 ***
Gender	−0.17869 ***	−0.18008 ***
Bergen	0.82545 ***	0.79042 ***
Aamot	1.15990 ***	1.10995 ***
Aardal	1.01430 ***	0.98399 ***
Halden	0.52663 ***	0.51371 ***
Harstad	0.80953 ***	0.78919 ***
Haugesund	−0.42288 ***	−0.44788 ***
Karasjok	1.59609 ***	1.61300 ***
Kragero	−0.16114 ***	−0.17753 ***
Kristiansund	0.47672 ***	0.38116 ***
Lenvik	1.02461 ***	1.00553 ***
Lillesand	−0.42022 ***	−0.44860 ***
Mandal	0.67481 ***	0.64102 ***
Meldal	0.22449 ***	0.18315 ***
Meloy	0.98197 ***	0.95077 ***
Modum	−0.17649 **	−0.22802 ***
Nord-Fron	0.70804 ***	0.67395 ***
Sauda	0.88510 ***	0.84392 ***
Sigdal	−0.22322 ***	−0.25864 ***
Stjordal	0.54919 ***	0.51972 ***
Stokke	−0.20470 ***	−0.21603 ***
Voss	0.34238 ***	0.31988 ***
Oslo × Year	0.22655 ***	0.27675 ***
Bergen × Year	0.14077 ***	0.18696 ***
Aamot × Year	0.09997 ***	0.13772 ***
Aardal × Year	0.24611 ***	0.27352 ***
Halden × Year	0.18834 ***	0.22655 ***
Harstad × Year	0.19484 ***	0.23181 ***
Haugesund × Year	0.56615 ***	0.62424 ***
Karasjok × Year	0.24142 ***	0.29275 ***
Kragero × Year	0.49890 ***	0.53762 ***
Kristiansund × Year	0.12880 **	0.20916 ***
Lenvik × Year	0.21795 ***	0.26162 ***
Lillesand × Year	0.29705 ***	0.34212 ***
Mandal × Year	0.25039 ***	0.28060 ***
Meldal × Year	0.17480 ***	0.22898 ***
Meloy × Year	0.18384 ***	0.21733 ***
Modum × Year	0.23927 ***	0.30226 ***
Nord-Fron × Year	0.23307 ***	0.26633 ***
Sauda × Year	0.31104 ***	0.34489 ***
Sigdal × Year	0.07901 *	0.11065 **
Stjordal × Year	0.16461 ***	0.20958 ***
Stokke × Year	0.32892 ***	0.36146 ***
Voss × Year	0.23107 ***	0.26995 ***

Notes: ***** *p* < 0.05, ****** *p* < 0.01, ******* *p* < 0.001; 564,738 Female = 1, Male = −1; Reference: Oslo.
